# Dietary resveratrol on growth performance, serum biochemical parameters, antioxidant capacity, and meat quality in broilers

**DOI:** 10.3389/fvets.2026.1857837

**Published:** 2026-07-27

**Authors:** Huaijin Xiang

**Affiliations:** School of Biology and Biological Engineering, South China University of Technology, Guangzhou, China

**Keywords:** abdominal fat, antioxidant capacity, growth performance, indigenous broiler, resveratrol

## Abstract

Indigenous broiler breeds are increasingly valued for their unique organoleptic attributes, although they typically exhibit suboptimal growth performance and a high propensity for abdominal fat deposition. This study evaluated the effects of dietary resveratrol (0, 250, and 1,000 mg/kg) on the growth performance, carcass traits, and antioxidant status of 252 one-day-old Wenshi Tianlu Black Five broilers over a 51-day period. Resveratrol supplementation increased significantly the average daily gain and decreased the feed-to-gain ratio across the entire trial (*p* < 0.01) without affecting feed intake. Specifically, 250 mg/kg resveratrol reduced the abdominal fat percentage (*p* < 0.05), while 1,000 mg/kg resveratrol decreased malondialdehyde level (*p* < 0.05) and increased the content of total cholesterol in serum (*p* < 0.01). However, supplementation of 250 mg/kg or 1,000 mg/kg resveratrol showed limited effects on the meat quality (*p* > 0.05). In conclusion, 250 mg/kg of resveratrol can improve carcass traits, while 1,000 mg/kg of resveratrol showed better effect of the growth performance and antioxidant ability in Wenshi Tianlu Black Five broilers.

## 1 Introduction

With the continuous elevation of global living standards, consumer demand for poultry has transitioned from mere quantity assurance toward a pursuit of superior quality and exquisite flavor. Wenshi Tianlu Black Five broilers, a typical premium local breed in Southern China, have gained increasing market favor due to their moderate growth cycle, delicious meat, and rich flavor (1). However, unlike modern fast-growing white-feathered broilers, local breeds are physiologically characterized by slower growth rates and a propensity for excessive abdominal fat deposition (2). Excessive fat accumulation not only impairs carcass traits and feed conversion efficiency but also often correlates with intense metabolic activity (3, 4). During the practical rearing process, local broilers are vulnerable to oxidative stress induced by environmental factors, handling, and transportation (5, 6). Oxidative stress triggers the excessive production of reactive oxygen species (ROS), which attack polyunsaturated fatty acids in cell membranes, resulting in lipid peroxidation products such as malondialdehyde (MDA) (7, 8). This oxidative damage compromises muscle cell integrity, leading to deteriorated meat color, increased drip loss, and an abnormal decline in pH, thereby undermining the inherent quality advantages of premium broilers (8). Therefore, identifying nutritional interventions to reduce abdominal fat deposition, accelerate growth, and enhance antioxidant capacity while maintaining superior flavor has become a critical challenge for the high-quality broiler industry (9).

Resveratrol (RES) is a non-flavonoid polyphenolic compound first isolated from the roots of Veratrum grandiflorum by Japanese scholar Takaoka in 1939 (10). Ubiquitously present in over 70 plant species, including grapes, *Polygonum cuspidatum*, mulberries, and peanuts, it acts as a phytoalexin produced by plants in response to environmental stressors such as ultraviolet radiation or fungal infection (11, 12). Renowned for its cardiovascular protective effects highlighted in the “French Paradox,” RES has garnered extensive attention in the pharmaceutical and healthcare sectors (13). Multiple studies have demonstrated that RES possesses exceptional biological functions, including antioxidant, anti-inflammatory, anti-tumor, and anti-aging properties (14, 15). As a safe, green, and efficient plant-derived functional additive, RES is increasingly utilized in livestock and poultry production. In broiler production, RES has been shown to significantly improve growth performance, promote immune organ development, and enhance carcass quality (16, 17). It may modulate lipid metabolism by activating the SIRT1/AMPK signaling pathway to downregulate key enzymes such as fatty acid synthase (FAS), thereby inhibiting excessive abdominal fat deposition (18); and improve antioxidant ability by directly scavenging superoxide anion radicals or enhancing the activity of antioxidant enzymes like superoxide dismutase (SOD) via the Nrf2 signaling pathway (19), which can reduce the level of MDA and maintain muscle fiber integrity, ultimately improving meat color and water-holding capacity.

Consequently, this study aimed to provide a reference for the application of RES in broiler nutrition by investigating the effects of varying dietary levels of RES on the growth performance, serum biochemical parameters, antioxidant capacity, and slaughter performance of broilers.

## 2 Materials and methods

### 2.1 Experimental design and diets

By using a single factor randomized controlled design, 252 healthy one-day-old Wen’s Tianlu Black Five cockerel broilers with similar body weight were randomly assigned into three groups and fed a basal diet (CON), a basal diet containing 250 mg/kg RES (purity≧95%) (RES250) or a basal diet containing 1,000 mg/kg RES (RES1000), respectively. Each group consists of 6 repetitions, and there were 14 broilers in each repetition. The experimental period lasted for 51 days.

### 2.2 Management and environment

Before the trial, the facility, including the floor, drinkers, and feeders, was thoroughly cleaned and disinfected. The broilers were reared on the floor, and each replicate was separated by wire mesh. Artificial lighting was controlled throughout the trial: 23 h of light per day during the first week, gradually decreasing to 18 h by the fourth week, and maintained at 18 h until the end of the experiment. The ambient temperature was initially set at 37 °C and decreased by 0.4 °C daily until it reached 25 °C, where it remained stable. The trial was divided into two phases: the starter phase (1–21 d) and the grower phase (22–51 d). All Broiler had ad libitum access to feed and water and were immunized according to routine procedures. Two different types of diets (starter diet for 0–21 d and finisher diet for 22–51 d) were considered to meet the nutrient levels followed by NRC (1994) (Supplementary Table S1), and the RES was mixed well in the feed.

### 2.3 Sample collection

At the end of the 51-day trial, one broiler per replicate with a body weight close to the average of the group was selected. After a 12-h fast (with access to water), the birds were weighed to determine the live body weight and then euthanized by pulling on the head while applying a ventrodorsal rotational force to the skull until an obvious reduction of resistance. Blood samples were collected using non-anticoagulant tubes. After standing for 30 min, the blood was centrifuged at 1500 × g for 10 min. The serum was then collected and stored for further analysis. After euthanasia, the carcasses were processed by removing feathers, pedal keratin, claw shells, and beak shells before weighing and dissection.

### 2.4 Growth performance and organ indices

Broilers were weighed by replicate on days 21 and 51 after fasting to calculate the Average Daily Gain (ADG). Feed consumption and residual feed were recorded per replicate to calculate the Average Daily Feed Intake (ADFI) and the Feed-to-Gain ratio (F/G). The liver, spleen, thymus, bursa of Fabricius, and abdominal fat were separated and weighed to calculate the organ indices using the following formula Organ Index (g/kg) = Organ weight (g)/ Live body weight (kg).

### 2.5 Slaughter performance

Slaughter performance was determined according to the ‘Performance terminology and measurements for poultry’ (NY/T 823—2020). Dressing weight: The weight of the carcass after bleeding, feathering, and removal of pedal keratin, claw shells, and beak shells. Eviscerated weight: The dressing weight minus the weight of the trachea, esophagus, crop, intestine, spleen, pancreas, gallbladder, heart, liver, proventriculus, gizzard, lungs, abdominal fat, head, shanks, and reproductive organs. Parameters including dressing percentage, eviscerated percentage, breast muscle percentage, leg muscle percentage, and abdominal fat percentage were calculated accordingly.

### 2.6 Meat quality

The pH of meat was measured at 24 h post-mortem using a portable pH meter (205, Testo, Germany). Three points were measured on both the breast and leg muscles to obtain the average. The lightness (L*), redness (a*), and yellowness (b*) were measured at three different positions on the breast and leg muscles using a portable colorimeter (NR20XE, 3nh, China), and the average values were recorded.

A 5 g meat sample of similar shape was taken from the breast and leg muscles, weighed, and placed in a drip loss tube at 4 °C for 24 h.


$$\text{Drip loss}\;(\%)=[\begin{array}{l} \left(\text{initial weight}-\text{weight after}\;24\;h\right)\\ /\text{initial weight} \end{array}]\times 100\%$$

Approximately 20 g of breast and leg muscle samples were weighed, vacuum-sealed in bags, and heated in a water bath until the core temperature reached 70 °C. After cooling and blotting surface moisture, the samples were reweighed.


$$\text{Cooking loss}\;(\%)=[\begin{array}{l} (\text{initial weight}-\text{cooked weight})/\\ \text{initial weight} \end{array}]\times 100\%$$

### 2.7 Serum antioxidant and biochemical parameters

Serum antioxidant indicators, including Total Antioxidant Capacity (T-AOC) and MDA were measured using commercial kits (Nanjing Jiancheng Bioengineering Institute, Nanjing, China) following the manufacturer’s instructions. Biochemical parameters, including Total Cholesterol (TC), Total Triglycerides (TG), Free Fatty Acids (FFA), Albumin (ALB), Globulin (GLB), Glucose (GLU), and Total Protein (TP), were determined using a Mindray BS-200 automated biochemical analyzer. The corresponding kits were purchased from Shenzhen Mindray Bio-Medical Electronics Co., Ltd.

### 2.8 Statistical analysis

Results are expressed as means ± standard deviation (SD). Statistical differences between groups were assessed using one-way analysis of variance (ANOVA), followed by *post hoc* analyses with Fisher’s least significant difference (LSD) test and Duncan’s multiple range test. *p* < 0.05 was considered statistically significant.

## 3 Results

### 3.1 Dietary supplementation of RES on the growth performance of broilers

The effect of RES on the growth performance of broilers is presented in Table 1. Dietary supplementation of RES showed no significant effect on the ADFI of broilers in starter phase (1–21 d) (*p* > 0.05). However, both 250 mg/kg and 1,000 mg/kg RES significantly increased the body weight at day 21 and ADG (*p* < 0.05). The RES250 group also showed a lower F/G than the control group (*p* < 0.05). In the grower phase (21–51 d), broilers in RES250 and RES1000 groups had significantly higher body weight at day 51 (*p* < 0.05) than the control, while the ADFI kept no change. The RES1000 group exhibited significantly greater ADG and the lowest F/G (*p* < 0.05), which was superior to both the RES250 and control groups (*p* < 0.05). Over the entire experimental period (1–51 d), supplementation of 250 mg/kg or 1,000 mg/kg showed no significant effect on overall ADFI (*p* > 0.05), but increased ADG and decreased F/G (*p* < 0.05).

**Table 1:** The effect of RES on the growth performance of broilers.

Indexes	CON	RES250	RES1000	*p*-value
1-21d				
Initial BW (g)	43.57 ± 0.90	43.21 ± 0.75	43.33 ± 0.58	0.713
Final BW (g)	291.62 ± 23.20^b^	342.16 ± 9.07^a^	325.91 ± 22.81^a^	0.001
ADG/(g/d)	11.81 ± 1.12^b^	14.24 ± 0.43^a^	13.46 ± 1.07^a^	0.001
ADFI/(g/d)	32.55 ± 2.96	32.17 ± 2.63	32.62 ± 1.91	0.947
F/G	2.77 ± 0.34^a^	2.26 ± 0.19^b^	2.44 ± 0.28^ab^	0.017
21-51d				
Initial BW	291.62 ± 23.20^b^	342.16 ± 9.07^a^	325.91 ± 22.81^a^	0.001
Final BW (g)	1056.99 ± 54.92^b^	1159.53 ± 65.68^a^	1167.33 ± 45.93^a^	0.006
ADG/(g/d)	25.51 ± 1.29^b^	27.25 ± 2.26^ab^	28.05 ± 0.97^a^	0.043
ADFI/(g/d)	107.85 ± 6.08	105.32 ± 11.96	99.71 ± 5.67	0.261
F/G	4.22 ± 0.11^a^	3.86 ± 0.28^b^	3.56 ± 0.21^c^	0.001
1-51d				
ADG/(g/d)	19.87 ± 1.07^b^	21.89 ± 1.29^a^	22.04 ± 0.90^a^	0.006
ADFI/(g/d)	70.20 ± 2.89	68.75 ± 6.98	66.17 ± 3.19	0.353
F/G	3.87 ± 0.12^a^	3.43 ± 0.24^b^	3.28 ± 0.21^b^	<0.001

CON, a basal diet; RES250, a basal diet containing 250 mg/kg RES. RES1000, a basal diet containing 1,000 mg/kg RES. Data are shown as means ± SD. In the same row, values with different small letter superscripts mean a significant difference (*p* < 0.05). The same as below.

### 3.2 Dietary supplementation of RES on serum biochemical parameters

As shown in Table 2, dietary supplementation of 250 mg/kg RES significantly increased the serum TP content (*p* < 0.05), while 1,000 mg/kg RES decreased the GLB content (*p* < 0.05). In terms of lipid metabolism, the serum TC in the RES1000 group was significantly higher than that in the control and RES250 groups (*p* < 0.05), while the contents of GLU, ALB, TG and FFA kept no significant change (*p* > 0.05).

**Table 2:** The effect of RES on serum biochemical parameters in broilers.

Indexes	CON	RES250	RES1000	*p*-value
GLU (mmol/L)	8.71 ± 0.89	8.83 ± 1.11	9.07 ± 1.02	0.826
TP (g/L)	23.72 ± 4.57^b^	28.73 ± 3.19^a^	21.09 ± 3.47^b^	0.001
ALB (g/L)	12.95 ± 0.96	15.23 ± 1.94	13.22 ± 2.20	0.085
GLB (g/L)	12.33 ± 2.55^a^	13.5 ± 4.30^a^	8.65 ± 1.24^b^	0.033
TG (mmol/L)	0.25 ± 0.04	0.27 ± 0.07	0.24 ± 0.05	0.666
TC (mmol/L)	3.34 ± 0.45^b^	4.09 ± 0.63^b^	5.20 ± 0.73^a^	0.001
FFA (mmol/L)	0.21 ± 0.04	0.18 ± 0.07	0.18 ± 0.04	0.464

### 3.3 Dietary supplementation of RES on the antioxidant capacity

The results for serum antioxidant capacity are summarized in Table 3. Dietary supplementation of RES showed no significant effect on the T-AOC (*p* > 0.05). However, 1,000 mg/kg of RES significantly reduced the level of MDA in serum (*p* < 0.05), while 250 mg/kg group showed no significant difference (*p* > 0.05), as compared to the control group.

**Table 3:** The effect of RES on antioxidant parameters in broilers.

Indexes	CON	RES250	RES1000	*p*-value
T-AOC (U/mL)	6.70 ± 1.55	7.06 ± 1.98	6.22 ± 1.5	0.699
MDA (nmol/mL)	4.51 ± 1.32^a^	3.56 ± 0.52^ab^	3.16 ± 0.48^b^	0.045

### 3.4 Dietary supplementation of RES on the slaughter performance

The slaughter performance data are shown in Table 4. Dietary supplementation of 250 mg/kg or 1,000 mg/kg RES had no significant effect on the dressing percentage, eviscerated percentage, or breast muscle yield (*p* > 0.05). However, the thigh muscle yield and abdominal fat yield in RES250 group were significantly lower than that in the control group (*p* < 0.05), while no significant difference was observed between the RES1000 group and the control group (*p* > 0.05).

**Table 4:** The effect of RES on the slaughter performance of broilers.

Indexes	CON	RES250	RES1000	*p*-value
Dressing percentage (%)	91.85 ± 2.29	91.50 ± 1.74	92.53 ± 3.04	0.761
Eviscerated carcass yield (%)	71.34 ± 8.37	76.66 ± 1.78	72.82 ± 6.92	0.352
Breast yield (%)	14.15 ± 4.75	11.37 ± 2.49	11.43 ± 3.68	0.365
Thigh yield (%)	19.22 ± 2.61^a^	15.94 ± 1.05^b^	17.41 ± 1.98^ab^	0.038
Abdominal fat yield (%)	1.11 ± 0.38^a^	0.63 ± 0.18^b^	1.39 ± 0.44^a^	0.007

### 3.5 Dietary supplementation of RES on the meat quality

The effects of RES on the meat quality of breast and thigh muscles are presented in Table 5. In breast muscle, supplementation of 250 mg/kg or 1,000 mg/kg RES showed no significant effect on the lightness (L*), redness (a*), yellowness (b*), cooking loss and pH at 24 h (*p* > 0.05), but significantly decreased the drip loss (*p* < 0.05), as compared to the control group. In thigh muscle, no significant differences were observed in redness, yellowness, drip loss, cooking loss, and pH at 24 h (*p* > 0.05), but both 250 mg/kg and 1,000 mg/kg RES increased the lightness (*p* < 0.05).

**Table 5:** The effect of RES on the meat quality of broilers.

Indexes	CON	RES250	RES1000	*p*-value
Breast muscle				
L*	45.60 ± 3.32	47.09 ± 2.49	49.10 ± 8.82	0.569
a*	3.86 ± 1.28	5.56 ± 1.96	5.18 ± 3.00	0.380
b*	5.84 ± 1.72	7.98 ± 2.29	6.59 ± 3.33	0.359
Drip loss (%)	2.57 ± 0.56^a^	1.45 ± 0.43^b^	1.07 ± 0.43^b^	0.001
Cooking loss (%)	19.43 ± 3.74	20.35 ± 3.46	20.17 ± 2.63	0.888
pH at 24 h	5.89 ± 0.16	5.94 ± 0.19	5.89 ± 0.14	0.830
Thigh muscle				
L*	43.64 ± 7.78^b^	50.27 ± 2.46^a^	51.66 ± 3.30^a^	0.034
a*	10.05 ± 2.96	10.16 ± 2.83	7.24 ± 2.96	0.180
b*	7.61 ± 1.75	9.49 ± 1.99	8.57 ± 2.07	0.275
Drip loss (%)	1.13 ± 0.31	1.01 ± 0.46	1.28 ± 0.35	0.520
Cooking loss (%)	21.27 ± 7.25	21.79 ± 4.55	19.66 ± 6.29	0.837
pH at 24 h	6.18 ± 0.13	6.24 ± 0.26	6.30 ± 0.30	0.704

### 3.6 Dietary supplementation of RES on the organ indices

The organ indices are shown in Table 6. Dietary supplementation of 250 mg/kg or 1,000 mg/kg RES showed no significant impact on the indices of liver, thymus and bursa (*p* > 0.05). However, the spleen index in RES250 group was significantly higher than that in the control and RES1000 group (*p* < 0.01).

**Table 6:** The effect of RES on organ indices in broilers.

Indexes	CON	RES250	RES1000	*p*-value
Liver (%)	2.12 ± 0.14	2.23 ± 0.15	2.05 ± 0.14	0.126
Spleen (%)	0.23 ± 0.10^b^	0.38 ± 0.08^a^	0.23 ± 0.05^b^	0.009
Thymus (%)	0.19 ± 0.06	0.17 ± 0.04	0.18 ± 0.03	0.827
Bursa (%)	0.34 ± 0.06	0.32 ± 0.09	0.29 ± 0.05	0.450

## 4 Discussion

As a premium indigenous breed in Southern China, Wen’s Tianlu Black Five broilers are characterized by relatively slow growth rates, a pronounced tendency toward abdominal fat deposition, and an extended rearing cycle. These traits render them susceptible to oxidative stress triggered by environmental challenges, handling, and transportation (20, 21). In contrast to fast-growing white-feathered broilers, this breed exhibits greater metabolic flexibility, thereby providing more scope for nutritional modulation (22).

The present study demonstrated that dietary supplementation of RES significantly increased ADG and decreased F/G over the entire 51-day period, whereas ADFI kept no change. These findings indicate that RES enhances growth primarily by improving nutrient utilization efficiency rather than by stimulating feed intake (23, 24). Previous reports have shown that RES can improve intestinal morphology and reinforce intestinal barrier function, thereby increasing the absorptive surface area (25). However, most of those studies employed challenge models involving lipopolysaccharide, pathogenic bacteria, or coccidia (23, 25). The growth-promoting effects observed here under unchallenged conditions suggest that RES can improve feed efficiency without requiring an inflammatory or pathological challenge (20).

Abdominal fat percentage is a critical determinant of carcass quality and feed efficiency in broilers. In the current trial, supplementation of 250 mg/kg RES was associated with a reduction in abdominal fat percentage, which is consistent with earlier findings in white-feathered broilers and confirming the potential of RES for improving carcass traits in high-quality indigenous breeds (9). Mechanistically, RES activates the SIRT1/AMPK signaling pathway, which downregulates sterol regulatory element-binding protein-1c (SREBP-1c) and its downstream targets, including FAS and acetyl-CoA carboxylase (ACC). This coordinated suppression inhibits *de novo* lipogenesis and promotes *β*-oxidation of fatty acids, thereby redirecting energy toward muscle accretion rather than fat storage (22). Notably, the anti-adipogenic effect disappeared in the RES1000 group, indicating 1,000 mg/kg of RES may have adverse metabolic implications. The concomitant elevation of serum TC in the RES1000 group suggests that excessive RES may alter lipid transport or redistribution, ultimately diminishing the fat-reducing benefit when the optimal dosage threshold is exceeded (9, 26).

Oxidative stress constitutes one of the primary factors responsible for post-mortem meat quality deterioration in broilers. In this study, 1,000 mg/kg RES significantly lowered serum MDA concentration, although the T-AOC was unaltered. This reduction in lipid peroxidation indicates that RES exerts a specific antioxidant effect, even in the absence of a measurable change in overall T-AOC (27, 28). In addition, both 250 mg/kg and 1,000 mg/kg RES increased the lightness in thigh muscle, but showed limited effects on other indices of meat quality. These observations highlight a complex trade-off between growth promotion and meat quality optimization, warranting further histomorphological analysis of muscle fibers (20).

## 5 Conclusion

In conclusion, under the conditions of this trial, dietary supplementation of 250 mg/kg or 1,000 mg/kg RES improved growth performance and feed efficiency in Wenshi Tianlu Black Five broilers. Supplementation of 250 mg/kg RES appeared to reduce abdominal fat deposition, while 1,000 mg/kg RES showed relatively better antioxidant protection. Further studies with larger sample sizes and more diverse doses are needed to confirm these observations and elucidate underlying mechanisms.

## Data Availability

All data generated or analyzed during this study are available from the corresponding author upon reasonable request.

## Glossary

ACC: Acetyl-CoA carboxylase
ADG: Average Daily Gain
ADFI: Average Daily Feed Intake
ALB: Albumin
ALP: Alkaline Phosphatase
ALT: Alanine Aminotransferase
AMPK: AMP-activated protein kinase
ANOVA: Analysis of Variance
AST: Aspartate Aminotransferase
a*: Redness
b*: Yellowness
BW: Body Weight
CON: Control group
F/G: Feed-to-Gain ratio
FAS: Fatty acid synthase
FFA: Free Fatty Acids
GLB: Globulin
GLU: Glucose
GSH-Px: Glutathione peroxidase
L*: Lightness
MDA: Malondialdehyde
Nrf2: Nuclear factor erythroid 2-related factor 2
RES: Resveratrol
ROS: Reactive Oxygen Species
SD: Standard Deviation
SIRT1: Sirtuin 1
SOD: Superoxide dismutase
SREBP-1c: Sterol regulatory element-binding protein-1c
T-AOC: Total Antioxidant Capacity
TC: Total Cholesterol
TG: Total Triglycerides
TP: Total Protein
T-SOD: Total Superoxide Dismutase
