# Supplementary material for: Dietary resveratrol on growth performance, serum biochemical parameters, antioxidant capacity, and meat quality in broilers
Source: Front Vet Sci. 2026 Jul 27;13:1857837. doi: 10.3389/fvets.2026.1857837 (PMC13454032; doi:10.3389/fvets.2026.1857837)
Supplement: Supplementary file 1 [file Table_1.docx]

Supplementary Table 1

| Names of Primers | primer sequence（5′-3′） |
| --- | --- |
| 43652-MYO7A-F | aacgtgaaaggtcctccctg |
| 43873-MYO7A-F | tgggtgtcccaaggggaatt |
| 45717-MYO7A-R | tttctggctgacagctgtgg |
| 45972-MYO7A-R | tcctccgagttccctggtac |
| MYO7A-mut-F | AGATCTTTCTGAAGgagatgccttccctgg |
| MYO7A-mut-R | ccagggaaggcatctcCTTCAGAAAGATCT |
| pcMINI-MYO7A-BamHI-F | GCTCGGATCCagcacggaaaattggggttc |
| pcMINI-MYO7A-XhoI-R | tttcCTCGAGctccgcgactcccgcaggcc |
| pcMINI-N-MYO7A-BamHI-F | GCTCGGATCCatgCTGTTCGACCGGCACCTGTG |
| pcMINI-N-MYO7A-XhoI-R | tttcCTCGAGcagccagaaatctagcacag |
| pcMINI-F | CTAGAGAACCCACTGCTTAC |
| pcMINI-R | GCCCTCTAGActggtcattccggctc |
| pcMINI-N-F | CTAGAGAACCCACTGCTTAC |
| pcMINI-N-R | GCCCTCTAGActggtcattccggctc |

**1.Primer design:**

Design two pairs of nested primers 43652-MYO7A-F and 45972-MYO7A-R, 43873-MYO7A-F and 45717-MYO7A-R, and perform nested PCR using normal human gDNA as a template.

**2.Fragment amplification:**

(1) Using the product of the second round of nested PCR as a template, a 980bp DNA fragment was amplified using primers with pcMINI-MYO7A-BamHI-F and pcMINI-MYO7A-XhoI-R.

(2) Using the product of the second round of nested PCR as a template, the left half of the 456bp DNA fragment was amplified using primers with pcMINI-MYO7A-BamHI-F and MYO7A-mut-R; a 547bp DNA fragment of the right half of pcMINI-MYO7A-mut was amplified by primers with MYO7A-mut-F and pcMINI-MYO7A-XhoI-R; Using a 1:1 mixture of these two DNA fragments as a template, a 973bpDNA fragment was amplified using primers with pcMINI-MYO7A-BamHI-F and pcMINI-MYO7A-XhoI-R.

(3) Using the product of the second round of nested PCR as a template, a 1382 bp DNA fragment was amplified using primers with pcMINI-N-MYO7A-BamHI-F and pcMINI-N-MYO7A-XhoI-R.

(4) Using the product of the second round of nested PCR as a template, the left half 738bp DNA fragment was amplified using primers with pcMINI-N-MYO7A-BamHI-F and MYO7A-mut-R; Using MYO7A-mut-F and pcMINI-N-MYO7A-XhoI-R as primers, the right half 667bp DNA fragment of pcMINI-N-MYO7A-mut was amplified; Using a 1:1 mixture of these two DNA fragments as a template, a 1375 bp DNA fragment was amplified using primers with pcMINI-N-MYO7A-BamHI-F and pcMINI-N-MYO7A-XhoI-R.
