# Supplementary material for: Dietary resveratrol on growth performance, serum biochemical parameters, antioxidant capacity, and meat quality in broilers
Source: Front Vet Sci. 2026 Jul 27;13:1857837. doi: 10.3389/fvets.2026.1857837 (PMC13454032; doi:10.3389/fvets.2026.1857837)
Supplement: Supplementary file 2 [file Table_2.docx]

**Supplementary Table 2 Whole-exome sequencing detail of the proband**

| **Detail** | **Proband** |
| --- | --- |
| Total（bp） | 127,815,308 (100%) |
| Duplicate（bp） | 38,043,710 (29.76%) |
| Mapped（bp） | 127,750,969 (99.95%) |
| Properly mapped（bp） | 126,960,564 (99.33%) |
| PE mapped（bp） | 127,697,902 (99.91%) |
| SE mapped（bp） | 106,134 (0.08%) |
| With mate mapped to a different chr | 544,126 (0.43%) |
| With mate mapped to a different chr ((mapQ>=5)) | 475,009 (0.37%) |
| Initial bases on target（bp） | 60,456,963 |
| Initial bases on or near target（bp） | 136,297,444 |
| Total effective yield (Mb) | 19,100.43 |
| Effective yield on target (Mb) | 12,807.50 |
| Fraction of effective bases on target（%） | 67.1% |
| Fraction of effective bases on or near target（%） | 88.5% |
| Average sequencing depth on target | 211.84 |
| Bases covered on target（bp） | 60,333,188 |
| Coverage of target region（%） | 99.8% |
| Fraction of target covered with at least 100x（%） | 76.3% |
| Fraction of target covered with at least 50x（%） | 91.0% |
| Fraction of target covered with at least 20x（%） | 97.3% |
| Fraction of target covered with at least 10x（%） | 98.8% |
| Fraction of target covered with at least 4x（%） | 99.5% |
| Total SNPs | 126895 |
| Novel SNPs | 834 |
| Total InDels | 15074 |
| Novel InDels | 1103 |
| Gender | Male |
